# Irreversible Swelling Behavior and Reversible Hysteresis in Chemically Crosslinked Poly(vinyl alcohol) Gels

**DOI:** 10.3390/gels4020045

**Published:** 2018-05-21

**Authors:** Keiichiro Kamemaru, Shintaro Usui, Yumiko Hirashima, Atsushi Suzuki

**Affiliations:** 1 Graduate School of Education, Yokohama National University, 79-2 Tokiwadai, Hodogaya-ku, Yokohama 240-8501, Japan; kamemaru@pen-kanagawa.ed.jp (K.K.); hirashima-yumiko-mc@ynu.ac.jp (Y.H.); 2 Graduate School of Environment and Information Sciences, Yokohama National University, 79-7 Tokiwadai, Hodogaya-ku, Yokohama 240-8501, Japan; usui.shintarou@ma.nichigo.co.jp

**Keywords:** poly(vinyl alcohol), chemical gel, microcrystallite, hydrogen bond, swelling behavior, hysteresis

## Abstract

We report the swelling properties of chemically crosslinked poly(vinyl alcohol) (PVA) gels with high degrees of polymerization and hydrolysis. Physical crosslinking by microcrystallites was introduced in this chemical PVA gel by a simple dehydration process. The equilibrium swelling ratio was measured in several mixed solvents, which comprised two-components: a good solvent (water or dimethyl sulfoxide (DMSO)), and a poor organic solvent for PVA. In the case of aqueous/organic solvent mixtures subjected to a multiple-sample test, the swelling ratio decreased continuously when the concentration of the organic solvent increased, reaching a collapsed state in the respective pure organic solvents. In the case of DMSO, starting from a swollen state, the swelling ratio rapidly decreased by between 15 and 50 mol % when the concentration of the organic compound increased in a single-sample test. To understand the hysteresis phenomenon, the swelling ratio was measured in a DMSO/acetone mixed solvent, starting from a collapsed state in acetone. The reversibility of swelling in response to successive concentration cycles between DMSO and acetone was examined. As a result, an irreversible swelling behavior was observed in the first cycle, and the swelling ratio in acetone after the first cycle became larger than the initial ratio. Subsequently, the swelling ratio changed reversibly, with a large hysteresis near a specific molar ratio of DMSO/acetone of 60/40. The microstructures were confirmed by Fourier transform infrared spectroscopy during the cycles. The irreversible swelling behavior and hysteresis are discussed in terms of the destruction and re-formation of additional physical crosslinking in the chemical PVA gels.

## 1. Introduction

Hydrogels are defined as three-dimensional, crosslinked polymer networks swollen in water, for which an infinitesimal change in environment can bring on a large volume change [1]. The backbone networks are usually formed through the chemical or physical crosslinking of polymers. In chemical hydrogels, polymer chains are connected by covalent bonds, whereas in physical hydrogels, the bonds are non-covalent. Among synthetic polymers, both chemical and physical hydrogels of poly(vinyl alcohol) (PVA) have been extensively studied for practical applications, owing to their low toxicity and high biocompatibility [2,3,4].

Physical PVA gels can be prepared easily by a freeze-thaw method [5,6,7] in water, or by a cast-drying method [8,9], starting from an aqueous PVA solution. Swelling and mechanical properties depend on the microcrystallites, which can be examined using X-ray diffraction (XRD) and Fourier transform infrared (FT-IR) spectroscopy [5,7,10]. On the other hand, chemical PVA gels can be obtained by crosslinking PVA in solution by irradiation with γ-rays or electron beams [11,12], or by chemical reaction using crosslinking agents [13,14].

The swelling behavior of chemical PVA gels slightly crosslinked by glutaraldehyde (GA) [14,15,16] in mixed solvents was reported. It was found that the microcrystallites could be formed in a chemical PVA gel with high degrees of polymerization and hydrolysis during a dehydration process, which was confirmed by XRD measurements and FT-IR spectroscopy [16,17]. The formation of microcrystallites was suppressed by increasing the degree of chemical crosslinking. The swelling ratio in pure water at room temperature depends strongly on the degree of chemical crosslinking, resulting from the destruction of microcrystallites. In the case of a lower degree of chemical crosslinking density, the gel swells to a size larger than the initial volume at gelation. Moreover, the swelling ratios of the chemical PVA gels with different degrees of polymerization and hydrolysis have been measured in mixed solutions of water and organic solvents. Chemical PVA gels with high degrees of polymerization and hydrolysis assume a swollen state both in pure water and pure dimethyl sulfoxide (DMSO) (good solvents for PVA) [18], whereas these gels collapse in acetone, methanol, or ethanol (poor solvents). In addition to these simple swelling-shrinking changes, the cosolvent and cononsolvent behaviors are observed, depending on the degree of hydrolysis, temperature, and the combination of solvents [15]. In these experiments of solvent dependence, mixed solvents were prepared at different concentrations, and the samples were immersed in the respective solvents using a multiple-sample test. In order to discuss swelling behavior however, solvent concentration should be changed using one sample (single-sample test), which has not been extensively reported so far. 

In this study, PVA gels with high degrees of polymerization and hydrolysis were prepared by chemically crosslinking PVA using glutaraldehyde. Physical crosslinking by microcrystallites was introduced into this chemical PVA gel by a simple dehydration process. The swelling behavior of this chemical gel with physical crosslinks was examined in a two-component solvent: one is a good solvent (water or DMSO), while the other is poor (organic compound) for PVA. When the dehydrated sample was immersed in a solvent, it absorbed the solvent, swelled, and remained in its equilibrium gel state, which resulted in the destruction of weak microcrystallites. Firstly, the swelling ratio in aqueous/organic solvent mixtures with different ratios was measured by the multiple-sample test. Secondly, in order to understand the reversibility of the swelling ratio by changing the solvent composition, the swelling behavior was examined in mixed solvents of DMSO and organic solvents by the single-sample test, starting from an equilibrium state in DMSO. Finally, to explore the possibility of realizing a volume phase transition, the swelling ratio was measured in a DMSO/acetone mixed solvent using a single-sample test as a typical example of a combination of good and poor solvents. The swelling behavior is discussed in terms of the network microstructure of the gel, which was examined here by FT-IR measurements.

## 2. Results and Discussion

### 2.1. Swelling Behavior in Aqueous/Organic Mixtures

**Figure 1:**
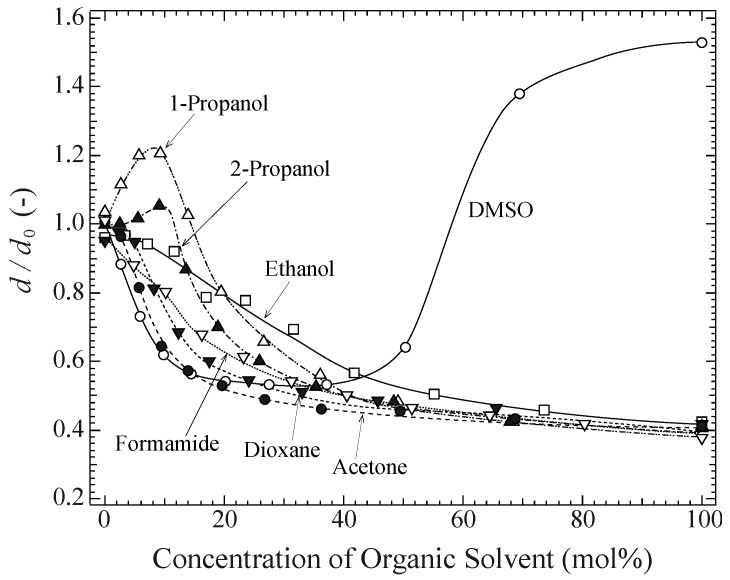
Swelling ratio of chemically crosslinked and dehydrated gels with different ratios of aqueous/organic solvent mixtures at room temperature (25 °C) measured by a multiple-sample test.

The swelling ratio, *d*/*d*_0_, of the chemical PVA gel in water was measured in several aqueous/organic solvent mixtures by a multiple-sample test. The gels were dehydrated after gelation and placed in their respective solvents. As shown in Figure 1, at lower concentrations of the organic solvents (alcohols, acetone, dioxane, formamide, ethanol, or DMSO) *d*/*d*_0_ was dependent on the solvent. At higher concentrations, *d*/*d*_0_ decreased continuously with an increase in concentration, reaching the collapsed state at 0.4 in the respective pure organic solvent (100 mol %), except in the case of DMSO. In the cases of propanols, *d*/*d*_0_ first increased and began to decrease from 10 mol %, before finally reaching a collapsed state. This exceptional change can be attributed to structural changes of bound water to the polymer [19]. The swelling behavior can be understood in terms of not only the solubility between the polymers and organic solvents, but also the formation and destruction of physical crosslinkings in the gels.

**Figure 2:**
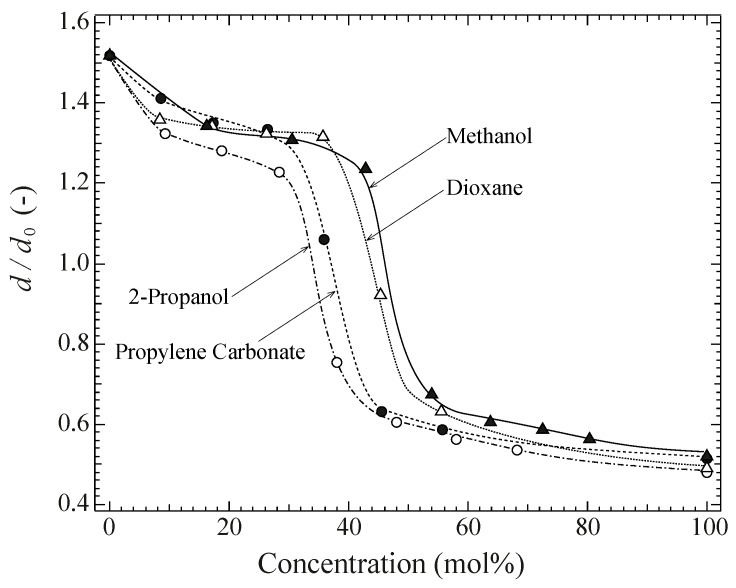
Swelling ratio of chemically crosslinked and dehydrated gels in DMSO-organic solvent mixtures with increasing concentration from single-sample tests.

It should be noted that the gels showed a collapsed swelling ratio of *d*/*d*_0_ = 0.4 in all the aqueous/organic solvent mixtures studied here, except for DMSO, i.e., for all two-component solvent mixtures containing a good solvent (water) and a poor solvent (organic). Considering that DMSO is a good organic solvent for PVA, and that *d*/*d*_0_ (=1.6) is much larger than that in pure water (*d*/*d*_0_ = 1.0), it would be interesting to measure *d*/*d*_0_ in a mixed solvent containing a poor organic solvent and DMSO. Starting from an equilibrium state in DMSO (organic solvent = 0 mol %), in which a dehydrated sample of the chemical PVA gel was placed, *d*/*d*_0_ was measured by a single-sample test. As is shown in Figure 2, *d*/*d*_0_ initially decreased gradually while increasing the organic solvent concentration, and it rapidly decreased at between 30 and 50 mol %. In order to examine the detailed behavior, *d*/*d*_0_ was measured by a single-sample test using DMSO/acetone as a typical example of the two-component system of organic solvent and DMSO; this has been reported in the next section.

### 2.2. Swelling Ratio in DMSO/Acetone Mixed Solvent

**Figure 3:**
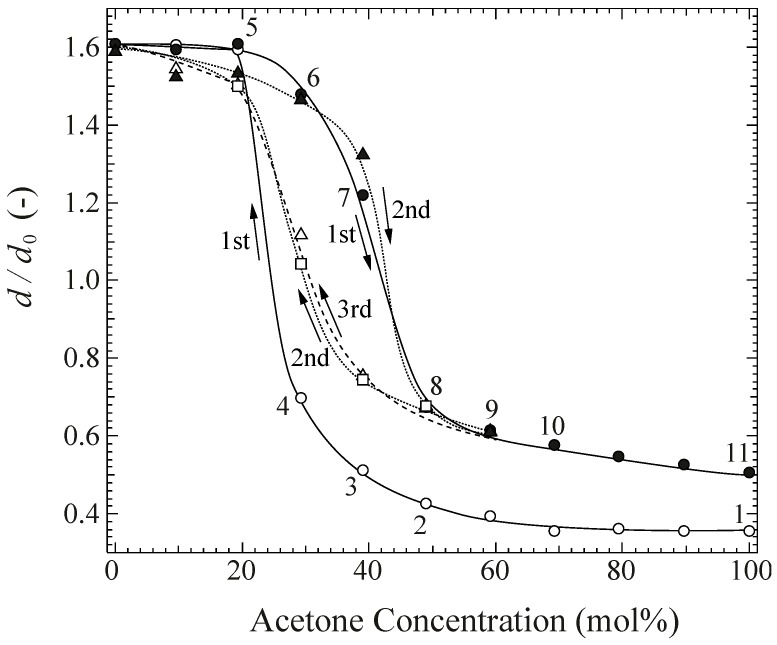
Swelling ratio of chemically crosslinked and dehydrated gels in a DMSO/acetone mixed solvent obtained by a single-sample test. The measurement was initiated from the collapsed state in acetone, and the concentration was successively changed relative to DMSO and acetone.

The equilibrium swelling ratio, *d*/*d*_0_ in a DMSO/acetone mixed solvent, was measured by varying successively the acetone concentration, which initially corresponded to the collapsed state of acetone (*d*/*d*_0_ = 0.4). Figure 3 shows the equilibrium *d*/*d*_0_ as a function of the acetone concentration at 25 °C. During the first decreasing process, where the acetone concentration was decreased, the system remained in the collapsed state above 70%, then it started to increase gradually, before becoming more rapid between 30 and 20 mol %, reaching the swollen state in pure DMSO. During the first increasing process, where the acetone concentration was increased again, *d*/*d*_0_ initially decreased gradually, and then rapidly between 40 and 50 mol %, and the gel returned to the collapsed state in pure acetone. As seen in this figure, an irreversible swelling behavior was observed, and the swelling ratio in acetone after cycling (*d*/*d*_0_ = 0.5) became larger than the initial ratio. Hereafter, the acetone concentration was again decreased (the second decreasing process) to that of pure DMSO, and increased continuously (the second increasing process), followed by the third decreasing process. The swelling ratio changed continuously and reversibly, with a large hysteresis at near the specific molar ratio of DMSO/acetone = 60/40.

The diameter change of the gels was continuous, and a large hysteresis was observed near a specific molar ratio. During the decreasing and increasing processes, the gels displayed reversible swelling behavior, except for the first decreasing process.

### 2.3. Change in Microcrystallites in the DMSO/Acetone Mixed Solvent

In order to discuss the effects of destruction and re-formation of microcrystallites on swelling behavior, ATR FT-IR spectra of the thin plate gel in the respective solvents were obtained during the first decreasing and successive increasing processes. The experimental conditions used for this thin plate gel were the same as those for the thin cylindrical gels used in the above diameter measurements.

**Figure 4:**
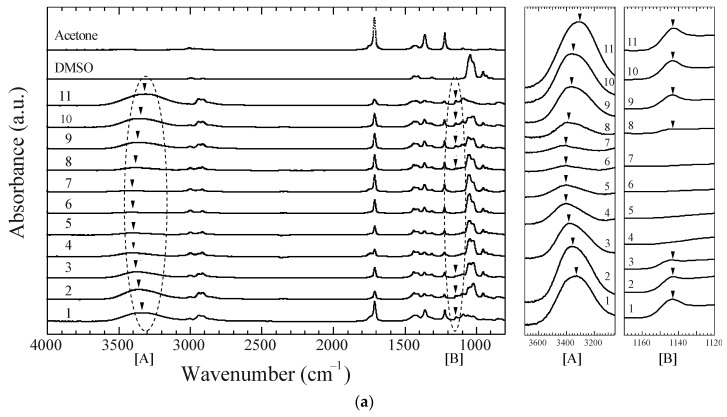

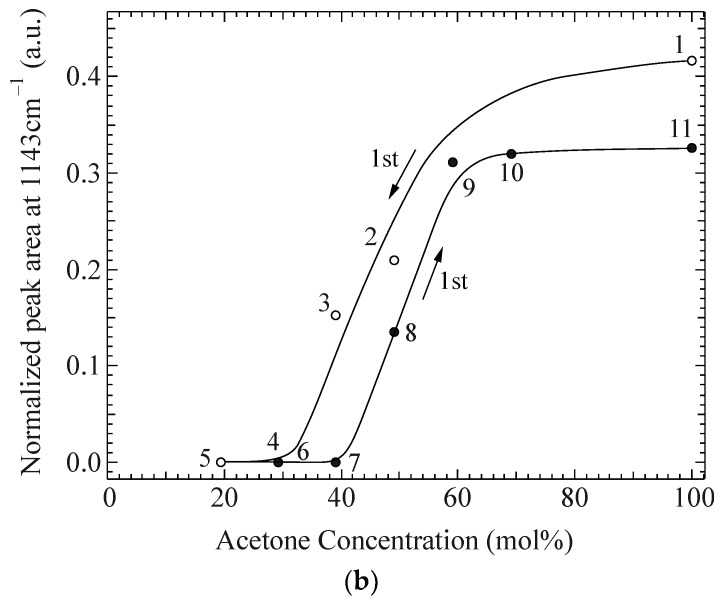
(**a**) ATR FT/IR spectra of a chemically crosslinked and dehydrated gel in various DMSO/acetone mixtures during the single-sample test shown in Figure 3. The detailed changes at around 3300 cm^−^^1^ (region [A]) and 1143 cm^−^^1^ (region [B]) are displayed; (**b**) The peak area at 1143 cm^−^^1^ normalized by the peak area at ca. 1427 cm^−1^, obtained by deconvoluting the spectra to remove the effects of other peaks. The respective number from 1 to 11 corresponds to those in Figure 3. The acetone concentrations are as follows; No. 1: 100, No. 2: 50, No. 3: 40, No. 4: 30, No. 5: 20, No. 6: 30, No. 7: 40, No. 8: 50, No. 9: 60, No. 10: 70, No. 11: 100 mol %.

Figure 4a shows the IR spectrum of gels with solvent during the first decreasing and successive increasing processes, which correspond to the processes shown in Figure 3. The spectra were normalized based on the C–H bending vibration at 1427 cm^−1^. Under these present experimental conditions, the peak area at ca. 1427 cm^−^^1^, which can be assigned to the –CH_2_ bending with the deformation bands of C–CH_3_ appearing at ca. 1377 cm^−^^1^ [20], was assumed to be equivalent in all spectra, and the spectra were normalized by the respective peak areas. The IR spectra of pure DMSO and acetone are added to this figure. The peaks located at ca. 1360 and 1210 cm^−1^ originate in the presence of acetone, while those at ca. 1050 and 900 cm^−1^ in the presence of DMSO. The large peak at around 3300 cm^−1^ consists of two components, which is assigned to O–H stretching of the non-hydrogen-bonded (3409 cm^−1^) and hydrogen-bonded O–H groups (3295 cm^−1^) [17]. As shown in this figure, the peak shifts to higher wavenumbers in the first decreasing process (No. 1 to 5), and to lower wavenumbers in the first increasing process (No. 5 to 11). The peak height shows a tendency to decrease and then increase during this cycle. These observations indicate that the ratio of the numbers of free to hydrogen bonded O–H groups increased and decreased, since the hydrogen bonds between polymer chains were respectively destroyed and re-formed by changing the DMSO/acetone ratio. On the other hand, the peak at 1143 cm^−1^ corresponding to C–O stretching due to the microcrystallites [21,22,23] disappears in the swollen state and re-appears in the collapsed phase. Figure 4b shows that the peak area at 1143 cm^−^^1^ was obtained by deconvoluting the spectra to remove the effects of the other peaks. The peak area at 1143 cm^−^^1^ decreases in the first decreasing process and increases in the successive increasing process, resulting from the decrement and increment of the ratio of microcrystallites to amorphous networks, or in other words, the decrement and increment in the degree of crystallization, respectively. There seems to be a discrepancy between the swelling ratio and peak area. For example, the peak area of No. 4 is almost zero in Figure 4b, although the corresponding swelling ratio does not reach the swollen state. This discrepancy might result from deficiencies in the experimental conditions, such as those due to the evaporation of solvent water at the surface. 

### 2.4. Origin of Reversible Change with a Large Hysteresis

Two important observations can be made about the observed swelling behavior of chemically crosslinked PVA gels in response to solvent concentration changes in DMSO/acetone mixed solutions. One is that there is an irreversible swelling behavior during the first decreasing and increasing processes. This irreversible swelling behavior suggests that microcrystallites are destroyed during the first decreasing process, and they were not completely re-formed during the first increasing process. In other words, microcrystallites can recover, but the recovered amount is smaller than the initial amount. According to a report on the ionized gels of poly(*N*-isopropylacrylamide) [24] and poly(sodium acrylate) [25], where re-swelling transitions were examined, hydrogen bonds were destroyed in response to the changes in the external conditions, and could not be reformed; furthermore, macroscopic volume changes were a one-way transition. In the case of the present PVA gels, the destruction of microcrystallites by increasing the temperature was reported earlier [16,17]. It was also a one-way transition, and re-formation of microcrystallites was not detected, although the swelling ratio decreased slightly when the temperature decreased. In the present case, the gels returned to a state close to the collapsed phase upon the re-formation of microcrystallites, which were detected for the first time. This evidence suggests that there are two kinds of microcrystallites: stable and unstable, i.e., reversible or irreversible changes in response to the change in solvent composition.

The other important observation is that *d*/*d*_0_ changed reversibly with a large hysteresis after the second decreasing process. The swelling behavior was reversible in the sense that *d*/*d*_0_ at high and low acetone concentration was the same in subsequent cycles, suggesting that the destruction and re-formation of microcrystallites that survived the first cycle were reversible in response to a change in solvent concentration. The large hysteresis is attributed to the formation of microcrystallites in the system; once the microcrystallites are destroyed during the decreasing process, subsequent increasing does not cause them to form until a much higher acetone concentration is used.

Finally, it should be noted that the destruction and re-formation of microcrystallites was detected in the present system, which resulted in the hysteresis phenomenon. Hysteresis can be found in many materials systems, such as ferromagnetic, ferroelectric, and viscoelastic materials. Comparing with those phenomena, the present hysteresis was observed in the equilibrium state, which is rate-independent, similar to magnetic hysteresis loops. In hydrogel systems, on the other hand, this phenomenon is reminiscent of multiple phase transitions [26], which are characterized by discontinuous and reversible changes in swelling ratios with large hysteresis, induced by a competitive balance between hydrogen bonds and hydrophobic interactions. Considering the hypothesis of universality of the phase transition of chemical gels, it might be possible to realize a reversible and discontinuous volume change in the present system. For that purpose, it would be necessary to adjust the material or environmental parameters to induce a positive osmotic pressure, and increase the swelling pressure in the good solvent relative to the destruction force of the microcrystallites. This remains a challenging topic in this field.

## 3. Conclusions

Chemical PVA gels were prepared by crosslinking PVA slightly with glutaraldehyde, washed with a large amount of pure water, and dehydrated fully in air at room temperature to introduce physical crosslinking. Both irreversible and reversible swelling behavior and hysteresis were observed in these PVA gels.

The gel reached its collapsed state in a pure organic solvent by a multiple-sample test, but did not reach the same state in a single-sample test. The equilibrium swelling ratios in a DMSO/acetone mixed solution were measured at room temperature to examine the relation between macroscopic swelling ratio and microscopic network structure. The swollen gel in DMSO could shrink in acetone, but did not return to the initial collapsed state after the gel had experienced the swollen state. This irreversible swelling behavior was attributed to the destruction of the microcrystallites formed during the initial dehydration after gelation. However, the swelling ratio changed continuously and reversibly, with a large hysteresis at around a specific molar ratio of DMSO/acetone of 60/40. This was consistent with the change in the microcrystallites observed by FT-IR measurements; microcrystallites were destroyed in DMSO and re-formed in acetone. This irreversible swelling behavior could not be observed in physical gels, although the sizes and amounts of microcrystallites were similar to those of the chemical gels. These observations were attributed to the irreversible destruction of weak microcrystallites, and to the reversible destruction and re-formation change of the strong microcrystallites.

Finally, it was concluded that the destruction and re-formation of microcrystallites in chemical PVA gels were reversibly controlled by concentration changes in the solvent, which was assisted by the chemical crosslinking. It is important to note that the present findings can be confirmed by other measurements, using XRD, DSC, and other techniques, which merit further research.

## 4. Experimental

### 4.1. Sample Preparation

Chemically crosslinked PVA gels with high degrees of polymerization and hydrolysis were obtained by crosslinking PVA solutions with GA. The PVA powder was supplied by Kuraray Co., Ltd. (Cat. No. PVA117, Tokyo, Japan), and was used without further purification. The average degree of polymerization was 1700, and the average degree of hydrolysis was between 98 and 99 mol %. The PVA powder was dissolved in deionized and distilled water at 90 °C or higher for more than 2 h. Gels were prepared in two shapes: thin cylindrical gels for the measurement of swelling ratio were prepared in glass microcapillaries, with an inner diameter of 1.44 mm and inner volume of 50 µL, and thin plate gels for FT-IR measurements were prepared between two slides with a thin spacer (thickness: about 2 mm). The concentration of the monomer units of PVA was fixed at ca. 2100 mM, and the proportion of GA (Wako Pure Chemical Industries, Ltd. (Tokyo, Japan), 25% aqueous solution) to the total concentration of monomer units of PVA plus the monomer of GA was 0.5 mol %. It is noteworthy that the degree of crosslinking of 0.5 mol % GA corresponds to a chain length of about 100 monomer units of PVA.

After mixing GA in dissolved PVA solution, 0.3 mL of 1 M HCl was added to the mixture as a catalyst. The solution was placed in a furnace at 30 °C for 48 h. After gelation, the cylindrical and plate gels were removed from the microcapillaries and mold, and subsequently washed in pure water (the water volume was 1000 times larger than the gel volume) for 48 h, to wash away any residual chemicals or uncrosslinked polymers from the polymer networks. Then, the gels were again placed in an oven at 30 °C for 48 h to dry.

### 4.2. Measurements of Swelling Ratio and FT-IR

The equilibrium diameter of a cylindrical gel, *d*, was measured by phase contrast microscopy. A sample was placed in respective solvents and left for more than 48 h, typically 96 h, which was sufficient for the gel to reach its equilibrium state. The swelling degree of the gel is represented by the swelling ratio, *d*/*d*_0_, where *d*_0_ is the gelation diameter (the inner diameter of the glass capillary, 1.44 mm).

To confirm the structural details of the gels, FT-IR measurements using the attenuated total reflectance (ATR) technique was performed on the dehydrated plate gels at room temperature. We used an FT-IR spectrophotometer (Jasco: FT/IR-610, Tokyo, Japan) equipped with an ATR (attenuated total reflectance) attachment with a ZnSe crystal. An appropriate amount of the gel was placed on the ZnSe crystal, and the FT-IR spectra were then recorded at room temperature.
